# Living in the New Normal: Effect of Residential Setting on Perception of a Meaningful Life among Older Women

**DOI:** 10.1093/geroni/igab046.3196

**Published:** 2021-12-17

**Authors:** Judith Scott, Helen Graham, Ann Mayo, Melissa Benton

**Affiliations:** 1 University of Colorado Colorado Springs, Colorado Springs, Colorado, United States; 2 University of San Diego, San Diego, California, United States

## Abstract

Perception of a meaningful life is related to depression, anxiety, and general well-being. The sense that one’s life is meaningful influences overall quality of life, which influences aging well. It is not clear whether differences in residential setting influence perception of a meaningful life. This study evaluated the effect of residential setting (community versus assisted living) on perception of a meaningful life in 48 older (79.7 ± 1.0 years) women living in the community (n=24) or assisted living (n=24) who were pair matched by age. They completed a one-time questionnaire regarding self-rated health and whether life has meaning. Both questions were scored on a 5-point scale with 0 indicating poor health or no life meaning and 4 indicating excellent health or strong life meaning. There were no significant differences in age between women in community living (CL) and assisted living (AL) (78.0 ± 09 vs. 81.5 ± 1.6 years, respectively; p=0.7). Both groups also reported similar self-rated health scores (CL: 2.4 ± 0.2; AL: 2.2 ± 0.2; p=0.4), indicating good-very good health. However, there were significant differences between groups in their perception of a meaningful life. Women in CL reported significantly lower scores compared to women in AL (2.9 ± 0.2 vs. 3.6 ± 0.1; p=0.006), indicating that women in CL perceived a less meaningful life. Based on our findings, it appears that the supportive infrastructure provided by AL residential settings may promote quality of life and successful aging by enhancing the perception of a more meaningful life.

